# New perspectives on VEGF signalling in Alzheimer's disease

**DOI:** 10.1111/bpa.70100

**Published:** 2026-04-12

**Authors:** Cherelle E. G. Emery, Seth Love, J. Scott Miners

**Affiliations:** ^1^ Cerebrovascular & Dementia Research Group, Learning and Research Building, Bristol Medical School, Translational Health Sciences University of Bristol Bristol UK

**Keywords:** Alzheimer's disease, PlGF, VEGF‐A, VEGF‐B, VEGFR1, VEGFR2

## Abstract

Vascular endothelial growth factor (VEGF) signalling mediates pleiotropic effects within the brain, encompassing angiogenesis, neuronal survival, and immune signalling. There is growing interest in the role of VEGF signalling in the pathophysiology of Alzheimer's disease (AD). The generation of single‐cell brain atlases and recent large multi‐omic studies, including analysis of CSF and bloods alongside post‐mortem brain tissue, have provided novel insights into the role of VEGF signalling in AD. Disruption of the VEGF‐A/VEGFR2 signalling pathway, due in part to elevated soluble VEGFR1 expression may contribute to pathogenic angiogenesis, BBB leakiness, and neuronal loss in AD. Induction of VEGF‐B/VEGFR1 signalling in microglia suggests that dysregulated VEGF‐mediated immune cell signalling is a further influence on AD pathogenesis. A reduction in expression of the ‘protective’ VEGFR3 and co‐receptors neuropilin 1 and 2 has also been recently linked to cognitive decline in AD. In large clinical studies, lower VEGF‐A levels in CSF and serum, raised soluble VEGFR1in CSF and elevated PlGF in CSF and serum, are predictive of more rapid cognitive decline and accelerated Alzheimer's disease neuropathological change (ADNC). This review discusses findings from recent multi‐omic studies of large clinical and neuropathological studies that prompt reconsideration of the nature of VEGF signalling in AD and shed light on some of the complexities and previous conflicts within the field.

## Graphical abstract

Emery et al. bring together findings from recent multi‐omic studies, including single‐cell mRNA analysis of human post‐mortem brain tissue, and proteomic analysis of matched CSF and blood samples in large clinical studies. The authors present evidence of the involvement of altered VEGF signalling in vascular and immune dysfunction and neurodegeneration in Alzheimer's disease, and consider the clinical diagnostic implications and the potential for novel therapies.

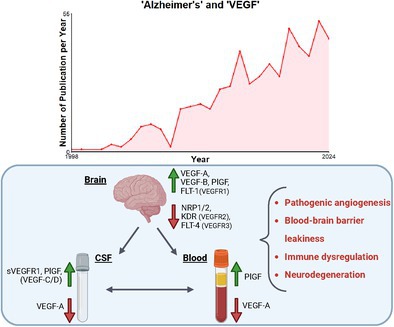

## 1 OVERVIEW

**FIGURE 1:**
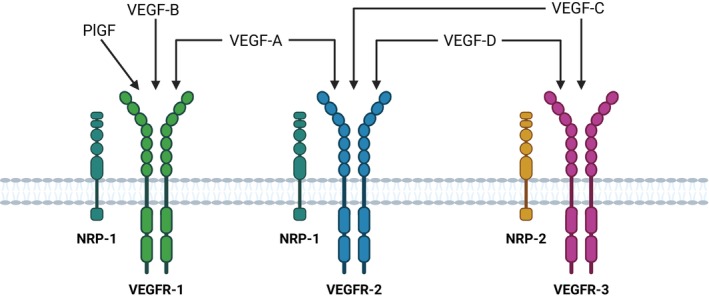
VEGF receptors, VEGFR1‐3, with their NRP co‐receptors and corresponding VEGF family ligands. NRP, neuropilin; PlGF, placental growth factor; VEGF, vascular endothelial growth factor; VEGFR, vascular endothelial growth factor receptor. Created with BioRender.com.

Vascular endothelial growth factors (VEGFs) are a family of homodimeric polypeptides comprising VEGFA‐D and placental growth factor (PlGF) [1]. VEGF family members signal through tyrosine kinase receptors—VEGFR1 (*FLT‐1*), VEGFR2 (*KDR*), VEGFR3 (*FLT‐4*)—and co‐receptors neuropilin (NRP) 1 and 2 [1, 2] (Figure 1). VEGF‐A predominantly signals via VEGFR1 and 2 on endothelial and neural cells; VEGF‐B and PlGF signal predominantly via VEGFR1 on neurons and microglia; and VEGF‐C and VEGF‐D via VEGFR3 play a key role in lymphangiogenesis [1, 3, 4].

Classical VEGF‐A/VEGFR2 signalling induces endothelial proliferation, migration and tip cell formation during angiogenesis, whilst VEGFR1 can act as a decoy receptor that binds and sequesters VEGF‐A [2, 5]. VEGF‐A/VEGFR2‐induced angiogenesis may improve brain perfusion in response to cerebral ischaemia; however, VEGF‐A/VEGFR2 signalling is also linked to pathogenic angiogenesis and blood–brain barrier (BBB) leakiness. Dysregulated VEGF‐A/VEGFR2 is implicated in cancer [6], diabetic retinopathy [7], macular degeneration [8], stroke [9] and AD [10, 11]. Targeting age‐related insufficiency in VEGF‐A/VEGFR2 signalling associated with impaired angiogenesis improves overall health and extends life‐span in rodents [12, 13].

VEGF‐A signalling via VEGFR2 is also neuroprotective, limiting tissue damage in neurodegenerative diseases, including AD [14]. The VEGF‐A/VEGFR2 pathway mediates long‐term potentiation (LTP) and induces the differentiation and migration of neural progenitor cells within an enriched environment [15] and in response to injury [16]. Ischaemic preconditioning protects both endothelial cells and neurons against subsequent severe ischaemia [17] and the protective effects of pre‐conditioning in conditions such as stroke or retinal ischaemia are likely due to the combined effects of VEGF‐A/VEGFR2 signalling in promoting angiogenesis and neuroprotection [18, 19].

The protective effects of VEGF signalling extend beyond the classical VEGF‐A/VEGFR2 pathway. VEGF signalling via VEGFR1 regulates chemotaxis and proliferation of microglia in vitro [20]. In a rat model of focal cerebral ischaemia, VEGF‐A released from injured neurons promoted M2 microglial polarization and contributed to overall neuroprotection following ischaemic pre‐conditioning [21]. VEGF‐C activation of VEGFR3 mediates M2 microglial polarization in vitro and improves motor deficits in a mouse model of traumatic brain injury (TBI) [22]. VEGF‐C/D/VEGFR3 signalling is also a major regulator of lymphangiogenesis in the brain [23].

**FIGURE 2:**
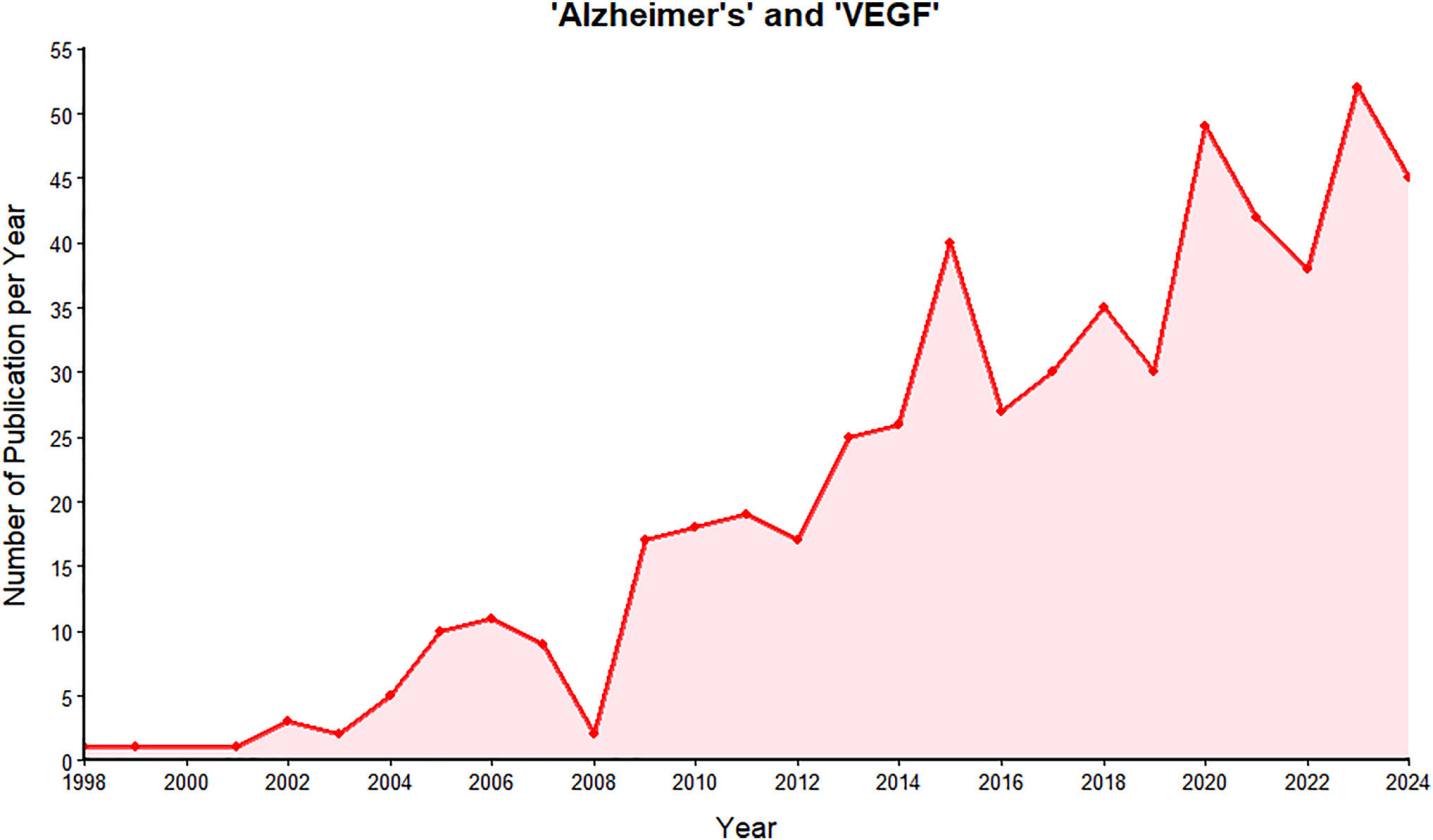
Graph showing the number of publications per year containing both ‘Alzheimer's’ and ‘VEGF’ in their title or abstract from 1998 to 2024. Data obtained from PubMed: https://pubmed.ncbi.nlm.nih.gov/.

Interest in VEGF signalling in AD continues to grow, as evidenced by the steady upward trend in the number of published papers since the turn of the century (Figure 2). Conflicting data from earlier studies hampered our understanding of VEGF signalling in AD, but recent multi‐omic single‐nucleus RNA (sn‐RNA) transcriptomic and proteomic analysis of human post‐mortem brain tissue, and assessment of VEGF trophic factors and receptors in CSF and blood, have provided novel insights and perspectives into VEGF signalling pathways in AD. These studies point towards an overall reduction in ‘protective’ VEGF‐A/VEGFR2 signalling and a disease‐related shift towards increased VEGF‐B‐ and PlGF‐mediated VEGFR1 signalling, associated with pathogenic angiogenesis, neuronal loss, and a dysregulated immune response, contributing towards cognitive decline and ADNC. An age‐related reduction in VEGF‐A expression in response to ischaemia [24], in addition to age‐related increased soluble VEGFR1 levels [25], may contribute to defective angiogenesis in AD. Age‐related defects associated with reduced VEGF‐A may be protected by lifestyle factors, for example, exercise is a potent stimulator of VEGF‐A and may counteract age‐related deficits in VEGF signalling and promote both angiogenesis [26] and neurogenesis [27].

Elevated soluble VEGFR1 in CSF, lower VEGF‐A in CSF and serum, and elevated PlGF in serum are all associated with dysregulated VEGF signalling in AD and have emerged as predictors of cognitive decline and progression of tau pathology in PET‐Aβ individuals. In this review, we provide an updated perspective on the role of the different VEGF ligand and receptor signalling pathways in AD, incorporating novel findings from recent multi‐omic analyses of human post‐mortem brain tissue, CSF and blood. We also direct the reader to relevant reviews that cover the broader aspects in VEGF‐A signalling in AD.

## 2 DYSREGULATED VEGF SIGNALLING IS RELATED TO COGNITIVE DECLINE AND NEUROPATHOLOGICAL CHANGE IN AD

VEGF‐A is expressed by multiple cell types in the CNS including endothelial cells, neurons, and astrocytes. VEGF‐A is upregulated in response to cerebral ischaemia via a HIF‐1α‐dependent mechanism, and signalling via endothelial VEGFR2 induces angiogenesis to restore blood flow [28]. Binding of VEGF‐A to VEGFR2 on endothelial cells also induces blood–brain barrier (BBB) breakdown [29, 30, 31]. Neuronal VEGF‐A/VEGFR2 signalling is neuroprotective [15]. VEGFR1 is an important modulator of VEGF‐A/VEGFR2 signalling, and soluble VEGFR1 can act as a decoy VEGF‐A receptor and block VEGFR2 signalling. Membrane‐bound VEGFR1 also serves as a partial agonist, regulating VEGFR2 signalling in a context‐dependent manner [32, 33]. The role of VEGF‐A/VEGFR2 signalling in angiogenesis and neuroprotection in the context of neurodegenerative diseases has been comprehensively reviewed [34, 35].

The importance of the VEGF‐A/VEGFR2 pathway in AD was confirmed in a gene‐set enrichment analysis of data from the Religious Orders Study and Rush Memory and Aging Projecy (ROSMAP) study (*n* = 634); among several hundred AD‐associated pathways, disrupted VEGF‐A/VEGFR2 signalling was the only pathway that correlated with all of the three phenotypes—amyloid, tau, and cognitive decline—that define AD [36]. Biochemical analysis has previously revealed that VEGF‐A levels are elevated in post‐mortem brain tissue in AD, the extent of the elevation correlating with the reduction in myelin‐associated glycoprotein (MAG):proteolipid protein‐1 (PLP1), this ratio being an indicator of the adequacy of ante‐mortem brain tissue oxygenation [37]. This was confirmed in independent studies of several brain regions including the frontal and parietal cortex. Reduced oxygenation of the precuneus, indicated by lower MAG:PLP1, was most pronounced in early‐intermediate AD pathology brains (Braak tangle stage III–IV) and correlated closely with elevated VEGF‐A. These findings provide further evidence that VEGF‐A is induced in response to cerebral hypoperfusion in the early stages of AD. VEGF‐A also correlated positively with the level of endothelin‐1, a potent vasoconstrictor peptide that is elevated in AD and upregulated in response to Aβ [38, 39, 40]. Reactive oxygen species‐associated release of endothelin‐1 from microglia and neurons, in response to oligomeric Aβ, induced pericyte mediated vasoconstriction of the microvasculature in slices of freshly resected human brain and in APP transgenic mice [41, 42]. VEGF‐A level was also elevated in mixed vascular‐AD dementia and related to subcortical white matter hypoperfusion [43]. A recent study of brain tissue from AD cases with little or no vascular co‐pathology revealed that VEGF‐A was elevated in multiple cortical and subcortical brain regions and correlated with the levels of CD31 (an endothelial marker and indicator of microvessel density) and fibrinogen (a marker of BBB leakiness), from an early stage of disease [44].

Despite the increase in VEGF‐A in AD brain tissue, there is a lack of consistent evidence of a corresponding increase in vascular density [45]. The level of VEGFR2, which is predominantly expressed by endothelial cells and astrocytes, was found not to be altered in AD, whereas soluble VEGFR1 showed a relative increase [46]. A relative shift towards VEGFR1 signalling in AD is likely to inhibit the angiogenic response to VEGF‐A and have a detrimental impact on neural cells. Administration of the humanized monoclonal anti‐VEGF‐A antibody bevacizumab to 5xFAD mice (which express mutant hAPP) restored the balance of VEGFR1 (lower) and VEGFR2 (higher) levels to normal, which prevented cerebrovascular leakage, restored vascular responsiveness to norepinephrine, and improved cognition [47]. Recent sn‐RNA sequencing studies in human brain tissue showed that the expression of *FLT‐1* (encoding VEGFR1) is induced in ‘pathogenic’ endothelial cells in AD [48, 49]. These data suggest that VEGF‐A levels are elevated in response to cerebral hypoperfusion in AD, but that a shift towards increased endothelial VEGFR1 expression contributes to pathogenic angiogenesis, associated with vascular remodelling and blood–brain barrier (BBB) leakiness from an early stage of disease [44].

VEGF‐A was shown to accumulate in proximity to Aβ plaques and to bind to Aβ peptides in vitro [50], suggesting that sequestration of VEGF‐A by plaques may reduce its bioavailability. The Aβ peptide also bound VEGFR2, preventing VEGF‐A‐mediated endothelial proliferation in vitro [51]. Aβ‐mediated sequestration of VEGF‐A, and inhibition of VEGFR2 signalling may, in combination with upregulated soluble VEGFR1 expression, lead to non‐productive angiogenesis in proximity to Aβ plaques in AD [52] (despite elevated levels of VEGF‐A in brain tissue). Binding of VEGF‐A to oligomeric Aβ also impaired long‐term depression (LTD) and LTP within the hippocampus of APP/PS1 mice, associated with defective VEGFR2 signalling [53].

Multi‐omic analysis of human post‐mortem brain tissue, combining bulk and sn‐RNA transcriptomic analysis and proteomic analysis within the same cohorts, has recently provided novel insights into cell‐specific alterations in VEGF signalling in AD. The studies have revealed that VEGF signalling pathways in addition to VEGF‐A/VEGFR2 are important in AD pathogenesis. In 2021, Mahoney et al. used bulk RNA sequencing to assess the gene expression of 10 VEGF family members in the dorsolateral prefrontal cortex from control (*n* = 180), MCI (*n* = 148) and AD dementia brains (*n* = 203), from participants in the ROSMAP study [54]. *VEGF‐B, PlGF*, *FLT‐1* (encoding VEGFR1) and *‐4* (encoding VEGFR3), were all upregulated in AD and associated with lower cognitive scores, more rapid cognitive decline, and more extensive Aβ and tau pathology; the findings were replicated in an independent cohort within the same study [54]. VEGF‐B and PlGF are major ligands for VEGFR1, which is predominantly expressed in neurons [46] and microglia [55]—the upregulation of VEGF‐B and PlGF is additional evidence of a shift towards VEGFR1 (and away from VEGFR2) signalling in AD.

In a follow‐up study, Seto et al., in 2023, confirmed increased gene expression of *VEGF‐B* and *FLT‐1* (encoding VEGFR1) in AD in the dorsolateral prefrontal cortex, posterior cingulate cortex, and caudate nucleus, across an expanded cohort (*n* = 531) [48]. Protein levels of VEGF‐B and VEGFR1, measured by mass spectrometry in the same sample set, were also elevated in AD and correlated strongly with cognitive decline and parenchymal Aβ load and tau tangle pathology. Sn‐RNA sequencing of cells from the dorsolateral prefrontal cortex in a subset of cases (*n* = 48) revealed elevated *VEGF‐B* and *FLT‐1* expression in microglia and oligodendrocytes, as well as endothelial cells [48]. Microglial expression of *VEGF‐B* and *FLT‐1* was strongly associated with cognitive decline, indicating that VEGF‐mediated immune cell activation is a key contributor to AD progression. The study also found reduced expression in AD of potentially ‘protective’ genes in the VEGF family: *NRP2, NRP1, FLT‐4* (encoding VEGFR3), in addition to confirming a reduction in *KDR* (encoding VEGFR2). Higher expression of *NRP2* was associated with better cognition and slower cognitive decline. Higher NRP1 protein levels were associated with better cognitive outcome and correlated inversely with Aβ plaque load. Sn‐RNA analysis revealed reduced *NRP1* expression in excitatory and inhibitory neurons in AD. *FLT‐4* expression was higher in AD and was associated with lower levels of Aβ and tau and with slower cognitive decline. In contrast, higher *KDR* (encoding VEGFR2) expression in inhibitory neurons was related to lower baseline cognitive score. These relationships were found within the caudate nucleus but did not apply to the prefrontal or cingulate cortex, indicating a complex, cell‐and region‐specific contribution of VEGF signalling in AD.

Wu and colleagues in 2025 performed a large sn‐RNA follow‐up study of 424 pre‐frontal cortex samples from ROSMAP [56]. Endothelial cells and microglia expressed higher levels of *FLT‐1* (VEGFR1) in AD, and this correlated with poorer baseline cognitive performance and accelerated cognitive decline in a subset of cases followed longitudinally [56]. Elevated expression of microglial *FLT‐1*, endothelial *FLT‐4* (VEGFR3), oligodendroglial *VEGF‐B*, astrocytic *VEGF‐D*, and oligodendrocyte progenitor cell *NRP1* was associated with elevated Aβ42 load. In contrast, higher *VEGF‐B* expression in inhibitory neurons was associated with less Aβ pathology, higher *NRP1* expression was related to lower parenchymal Aβ load, and higher astrocytic *NRP1* was related to lower tau tangle load. When all VEGF ligand‐receptor pairs among all cell types were summed, VEGF signalling overall was down‐regulated in AD. Network analysis of the sn‐RNA dataset showed that astrocytic *VEGF‐A* was the predominant ligand expressed, and that the top ligand ‘receiver’ cell‐type was excitatory neurons in controls, switching to endothelial cells in AD. Interestingly, *VEGF‐A*/*FLT1* signalling contributed to 9.5% of VEGF signalling in controls and increased to 13.8% in AD (likely to be mediated by upregulation of endothelial *FLT‐1* in AD).

Lau et al., in 2020, performed unbiased sn‐RNA analysis of 169,496 nuclei from the pre‐frontal cortex in AD (*n* = 12) and controls (*n* = 9) [49]. A higher proportion of endothelial cells was observed in AD (3%) compared to controls (1.2%). Expression of *FLT‐1* (VEGFR1) was upregulated within a subset of ‘angiogenic’ endothelial cells in AD. It may be relevant that oligomeric Aβ‐induced endothelial senescence is mediated by the induction of VEGFR1 [57]. In a recent study of human post‐mortem brain tissue, we found widespread elevation of VEGF‐A levels, associated with markers of neoangiogenesis and with BBB breakdown, from the early stages of AD [44]. The key role of VEGF‐A in neoangiogenesis and BBB breakdown was demonstrated in mice with experimental allergic encephalitis (an experimental model of multiple sclerosis), in which enrichment of endothelial tip cells, associated with neoangiogenesis, led to the formation of abnormal leaky blood vessels; these effects were prevented by administration of a VEGF‐blocking antibody [58].

**FIGURE 3:**
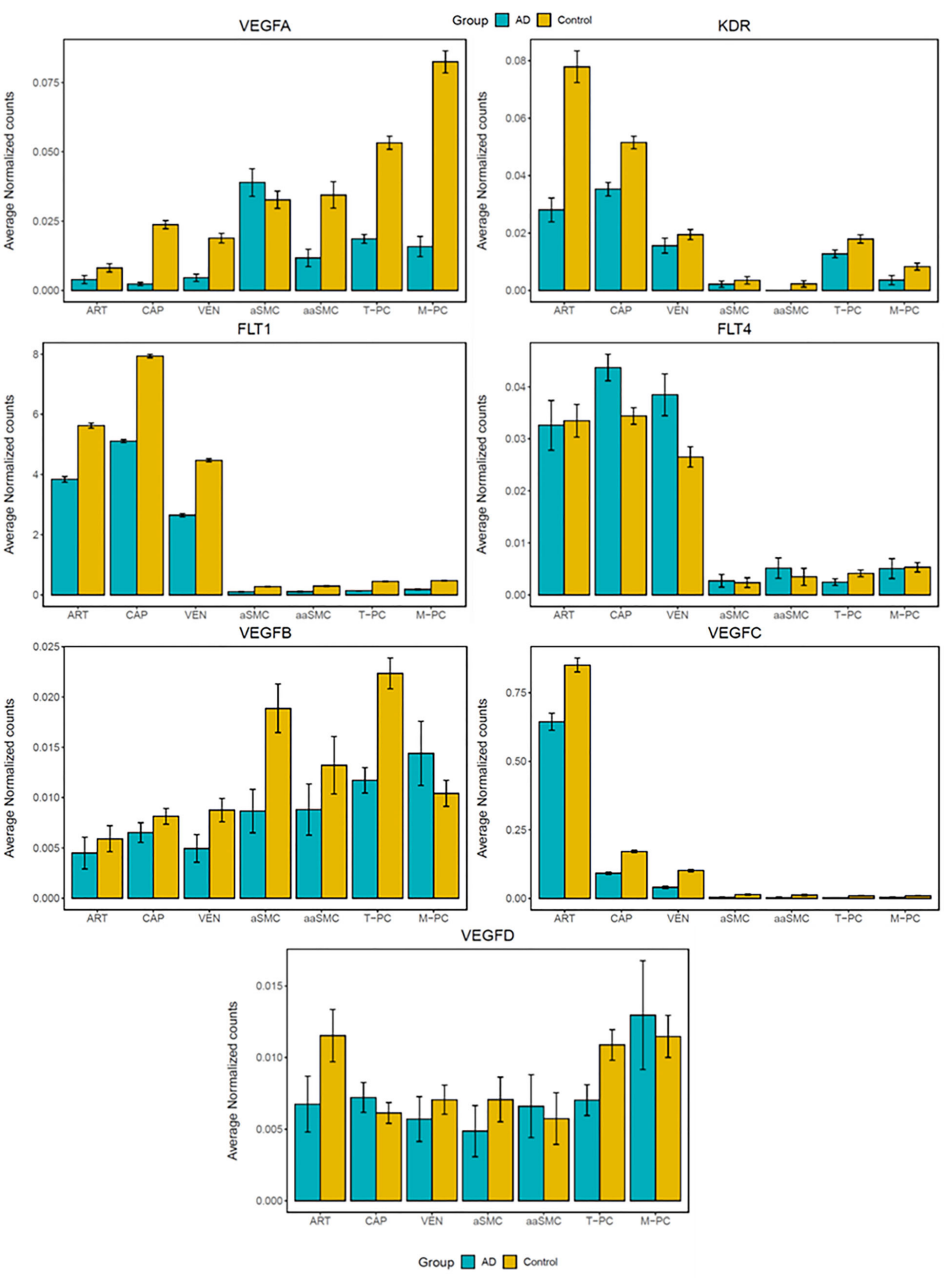
Analysis of VEGF family gene expression within vessel‐enriched preparations in Alzheimer's disease (AD). Analysis of the sn‐RNA dataset from the VINE‐Seq study. aSMC, vascular smooth muscle cell; aaSMC, arteriolar smooth muscle cell; ART, arterial endothelial cell; CAP, capillary endothelial cell; M‐PC, ECM‐regulating pericyte; T‐PC, solute transport‐pericyte; VEN, venous endothelial cell; P.FB, perivascular fibroblast. *Source*: https://twc‐stanford.shinyapps.io/human_bbb/.

Most sn‐RNA studies to‐date have not enriched for blood vessels and may have underestimated the contribution of VEGF‐A/VEGFR2 signalling in AD. Two recent sn‐RNA studies have been performed on vessel‐enriched populations of cells isolated from human post‐mortem brain tissue. Analysis of VEGF family gene expression profiles in 143,793 nuclei isolated from vessel‐enriched hippocampal and cortical samples from 9 AD and 8 control brains in the VINE‐Seq study [59], revealed a reduction in *VEGF‐A*/*KDR* (VEGFR2) expression in AD (Figure 3). All three VEGF receptors were predominantly expressed in endothelial cells—the expression of *FLT‐1*, as for *KDR*, was surprisingly lower in AD, whereas endothelial expression of *FLT‐4* (VEGFR3) was increased. The relative expression of *VEGF‐A* and *VEGF‐B* within vessel preparations was higher in controls, particularly within pericytes, suggesting that most of the increased *VEGF‐A* and *‐B* expression in AD may originate from non‐vascular cells, including neurons and glia.

Another sn‐RNA study in a vessel‐enriched sample of nuclei from frontal cortex also revealed reduced endothelial *VEGF‐A*/*KDR* expression in AD (*n* = 41) compared to non‐disease controls (*n* = 36) [60]. The authors proposed that despite the upregulation of genes that induce angiogenesis—including *HIF1A*, *ANGPT2*, and *FGF2*, it is the reduction in the endothelial *KDR* (VEGFR2) receptor expression that they hypothesised ultimately impairs VEGF signalling and blocks an effective angiogenic response in AD. A recent study of differentially expressed genes in the middle temporal gyrus in AD also found evidence of altered VEGF‐A/VEGFR2 signalling between pericytes and endothelial cells [61].

Single‐nuclei transcriptomic studies have also confirmed that VEGF signalling within the human brain extends beyond the canonical VEGF‐A/VEGFR2 pathway and involves a diverse range of CNS cells. There is an overall reduction in VEGF signalling in AD, which probably reflects impaired vascular VEGF‐A/VEGFR2 signalling and results in pathogenic angiogenesis, but there is also a shift towards overactivation of VEGF‐B and PlGF activation of VEGFR1 in endothelial cells and microglia that is closely related to cognitive decline and disease pathology (Aβ/tau) in AD [48, 56]. *FLT‐1* (VEGFR1) expression maps strongly to microglial cells (and oligodendrocytes to a lesser extent) in AD, suggesting that VEGF signalling regulates immune cell activation. VEGF‐A‐mediated *FLT‐1* (VEGFR1) signalling induces chemotaxis of microglia towards Aβ in vitro and in vivo, and double IF revealed co‐localisation of VEGF and VEGFR1 within Aβ plaques in AD brain tissue [55]. VEGF‐A activation of VEGFR1 was shown to induce the shedding of TREM‐2 via induction of ADAM10/17, promoting the phagocytosis of oligomeric Aβ [62]. VEGF‐A/VEGFR1 signalling in microglia would therefore be expected to facilitate Aβ clearance, and elevated VEGF‐B could competitively impair this protective response. The shift towards VEGF‐B/VEGFR1 signalling in AD may promote disease‐associated microglial activation (or impair their protective function) and stimulate pathogenic angiogenesis, contributing towards the disease‐associated phenotype observed in AD.

In sn‐RNA transcriptomic studies, *VEGFR3* receptor expression, and the expression of co‐receptors *NRP1* and *NRP2*, was negatively associated with cognitive decline and AD pathology [48, 56]. VEGF‐C binds to NRP‐2 and stimulates VEGFR3‐mediated lymphoangiogenesis, which may provide some protection in AD by facilitating Aβ clearance via the glymphatic system [63]. Higher VEGF‐B expression in inhibitory neurons and NRP1 expression in astrocytes was also related to lower Aβ and neurofibrillary tangle content, respectively [56]. VEGF‐B predominantly binds to VEGFR1 and NRP1 on neurons and has minimal effects on angiogenesis. Neuronal VEGF‐B/FLT‐1(VEGFR1) signalling was shown to stimulate adult neurogenesis [64] and to be neuroprotective [65, 66], rescuing neurons from apoptotic cell‐death [67].

**FIGURE 4:**
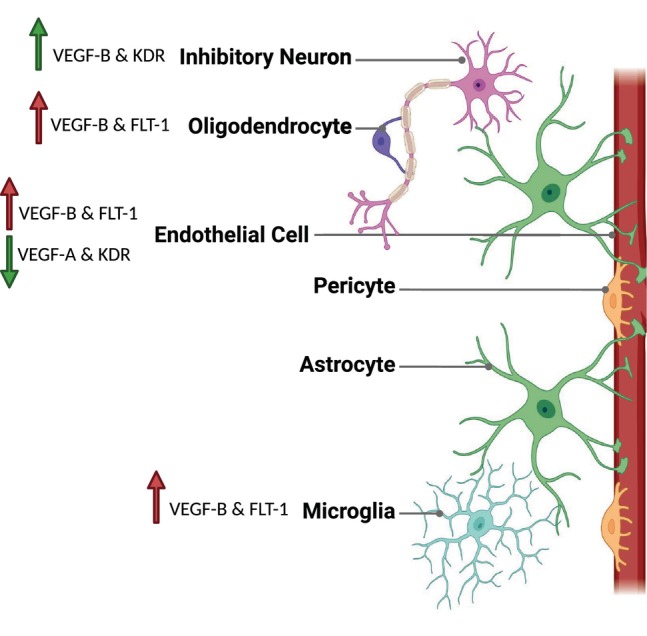
Summary of sn‐RNA analysis of cell‐type specific changes in VEGF family gene expression within human post‐mortem brain tissue in Alzheimer's disease. Relative changes in VEGF in specific cell populations indicated by red arrows (disease‐associated signalling) and green arrows (protective signalling), with the arrow direction corresponding to the change in gene expression. FLT‐1, vascular endothelial growth factor receptor 1 gene; KDR, vascular endothelial growth factor receptor 2 gene; VEGF, vascular endothelial growth factor genes. Created with BioRender.com.

The cell‐specific changes in VEGF family gene expression are summarised in Figure 4. These comprise a shift in the balance of VEGF signalling towards induction of VEGFR1 and overexpression of its ligands—PlGF and VEGF‐B and a concurrent reduction in VEGF‐A/VEGFR2 signalling. A shift towards pathogenic angiogenesis induced by activation of VEGFR1 signalling within endothelial and a reduction in protective VEGFR3 signalling, due in part to reduced expression of binding partners, NRP1 and NRP2. An overall shift in balance towards elevated VEGF‐B and PlGF signalling via VEGFR1 is likely to contribute to endothelial and neuronal damage, and the dysregulated microglial immune response observed in AD.

## 3 CSF AND BLOOD VEGF‐A AND VEGFR1 LEVELS ARE ASSOCIATED WITH COGNITIVE DECLINE AND ADNC

A large prospective longitudinal study by Hohman et al. in 2015 [68], found that CSF VEGF‐A did not differ significantly between cognitively unimpaired (*n* = 90), cognitively mildly impaired (*n* = 130) and AD (*n* = 59) participants selected from the Alzheimer's Disease Neuroimaging Initiative (ADNI) study. Lower baseline CSF VEGF‐A was, however, related to lower hippocampal volume in cross‐sectional analysis and accelerated hippocampal atrophy in a subset of participants followed longitudinally—this relationship was stronger in CSF AD biomarker+ve participants (i.e., with decreased CSF Aβ42 or increased tau). Lower CSF VEGF‐A was also associated with steeper cognitive decline in the Aβ+ve group. A follow‐up study by Tubi et al., in 2021 [69], revealed lower CSF VEGF‐A in Aβ+ve (*n* = 215) than Aβ‐ve (*n* = 95) individuals. The Aβ+ve individuals had lower MMSE scores and higher tau load. Within the Aβ+ve group, lower CSF VEGF‐A correlated with reduced cortical thickness and lower cognitive scores (ADNI‐EF and ANDI‐MEM). Together, these studies suggest that reduced CSF VEGF‐A levels are associated with AD pathology, cognitive decline, and neurodegeneration. Although VEGF‐A is elevated in brain tissue in AD, the CSF findings suggest that much of it may be sequestered, by sVEGFR1 (levels of which are elevated in AD [46]), oligomeric Aβ, or within Aβ plaques. Interaction of VEGF‐A with Aβ [70] may also influence cerebral amyloid angiopathy, and although direct evidence is currently lacking, this interaction has the potential to modulate vascular Aβ deposition in a manner similar to VEGF‐A deposition within parenchymal Aβ plaques [50].

Reduced serum VEGF‐A is also associated with cognitive decline in AD. This was first documented in 2007, in a comparison of AD patients (*n* = 51) with age‐and gender‐matched controls (*n* = 66) [71]. Yang et al., in 2024 [72], studied plasma levels of five VEGF family members, VEGF‐A, ‐C, ‐D, PlGF and FLT, by use of a V‐Plex angiogenesis kit, in 317 older adults from the Harvard Aging Brain Study. Baseline plasma levels were measured in CU participants who were followed over 12 years. Lower baseline plasma VEGF‐A was associated with accelerated cognitive decline in individuals who were Aβ PET+ve. Lower plasma VEGF‐A was also associated with elevated PET‐tau signal within the inferior temporal cortex. Mediation analysis revealed that the relationship between lower plasma VEGF‐A and cognitive decline in Aβ PET+ve individuals was almost completely mediated by changes in CSF tau and was independent of vascular pathology.

In a comparison between healthy controls (*n* = 29), amnestic MCI (*n* = 28), and AD (*n* = 31), serum VEGF‐A was significantly lower in AD than in amnestic MCI or healthy controls and was also lower in MCI compared to controls [73]. In this study, serum VEGF‐A level correlated inversely with Clinical Dementia Rating (CDR) score. A recent analysis of a pool of 30 plasma proteins also revealed that plasma VEGF‐A was lower in MCI (*n* = 6) than controls (*n* = 49) and rose significantly in AD (*n* = 52) [74]. Multivariate logistical regression modelling indicated that lower plasma VEGF‐A level was predictive of accelerated cognitive decline and correlated inversely with CDR score [74]. In a study of 72 older adults, Da Silva et al., in 2023, reported reduced plasma VEGF‐A in CDR 1 v 0, that is, in the early stages of AD [75]. In a recently published conference abstract, serum VEGF‐A was lower in MCI (*n* = 29) than in CU controls (*n* = 54). There was significant interaction between serum VEGF‐A level and Montreal Cognitive Assessment (MoCA) score that differed between controls and AD: serum VEGF‐A correlated negatively with MoCA score in healthy controls but positively with MoCA score in AD suggesting a relationship dependent on disease stage.

The level of CSF soluble VEGFR1 is elevated in MCI, as well as AD, and is related to changes in tau in PET‐Aβ+ve individuals. Analysis by Janelidze et al., in 2018 [77], of MCI and AD participants in the Swedish BioFINDER study, and of cognitively unimpaired (CU) participants recruited as part of the Malmö Diet Cancer Study, revealed that CSF VEGFR1 was elevated in MCI (*n* = 256) and AD (*n* = 57) compared to CU participants (*n* = 508). CSF VEGFR1 was higher in CU and MCI participants with reduced CSF Aβ42:40 (i.e., AD Aβ biomarker‐positive) and was strongly related to CSF t‐tau and p‐tau181 in Aβ biomarker+ve individuals. Higher baseline CSF VEGFR1 was an independent negative predictor of time to dementia, particularly in women, in a longitudinal subset of samples (*n* = 728) [77].

More recently, Winfree et al., in 2025 analysed CSF from participants in the Vanderbilt Memory and Aging Project (VMAP) [78]. CSF VEGFR1 was unchanged in the MCI group (*n* = 71) compared to CU controls (*n* = 82) but was significantly higher in MCI individuals who were tau+ve. Within the tau+ve group, VEGFR1 correlated with CSF Aβ40:42, p‐tau‐181 and p‐tau‐231. Despite interaction between VEGFR1, tau and cognition in Aβ+ve individuals, CSF VEGFR1 did not correlate with baseline cognition, longitudinal memory changes, or the CSF/plasma albumin ratio (Qalb)—a measure of BBB leakiness [78]. The study also found comparable plasma VEGFR1 in CU controls and MCI; however, higher plasma VEGFR1 was associated with increased plasma Aβ42 and p‐tau‐231.

The China Aging and Neurodegenerative Disease Initiative (CANDI) examined the CSF levels of VEGF signalling proteins in cases stratified by CDR score: CDR = 0 (*n* = 44), 0.5 (*n* = 60) and ≥1 (*n* = 96) [79]. Elevated CSF sVEGFR1 was associated with lower CSF Aβ42:40 and elevated CSF t‐tau and p‐tau^181^. Increased CSF VEGFR1 was also associated with increased white‐matter hyperintensities (WMH) and cortical atrophy. Levels of CSF sVEGFR2, VEGF‐C and ‐D, and PlGF were raised in CDR 0.5 (and further in CDR ≥1) and correlated with CSF tau [79]. CSF sVEGFR2 and PlGF levels correlated with Qalb. Higher CSF VEGF‐C was related to increased cortical thickness. In summary, elevated CSF levels of VEGF family proteins in these studies were strongly associated with markers of endothelial injury and pathogenic angiogenesis, and with tau pathology, brain atrophy, and cognitive decline, beginning in cognitively unimpaired Aβ+ve individuals.

Findings in CSF and serum have been inconsistent, possibly reflecting variation in methodology [80]. Studies mapping changes in CSF and serum to cognitive decline, and to the presence of Aβ and tau, β reveal that the nature of VEGF signalling changes during the evolution of AD; these changes may both influence and reflect the concomitant changes in Aβ and tau pathology during disease progression [81, 82]. Gao et al., in 2025, and Winfree et al., in 2025, reported that elevated CSF VEGFR1 was strongly associated with accelerated cognitive decline, tracking CSF tau closely, specifically in PET Aβ+ve individuals [78, 79]. These findings are in keeping with recent studies showing that CSF markers of endothelial and pericyte injury, including VEGFR1, ICAM, VCAM1, and sPDGFRB, are elevated in MCI and closely track changes in CSF tau in Aβ+ve participants [77, 83, 84]. Soluble VEGFR1 may sequester and inactivate VEGF‐A, which would otherwise induce an angiogenic response and offer neuronal protection. Measurement of circulating VEGF‐A, which is reduced from early in the development of AD, may be useful to predict cognitive decline and track the progression of tau pathology in PET‐Aβ+ve individuals [72].

## 4 ELEVATED CIRCULATING VEGF AND PlGF IS ASSOCIATED WITH WHITE MATTER INJURY RELATED TO SMALL VESSEL DISEASE AND LATE‐NC ASSOCIATED VASCULOPATHY

White matter injury caused by cerebral small vessel disease (cSVD) is the major pathological substrate of vascular cognitive impairment (VCI) and may contribute in up to 50% of patients with dementia. Although there are few direct studies, evidence suggests that dysregulated VEGF‐A signalling contributes to arteriosclerosis, pathogenic angiogenesis, BBB leakiness, and to recruitment and infiltration of immune cells within the perivascular space. cSVD is common in AD and accelerates neurodegeneration and cognitive decline (reviewed [85, 86] and see [87, 88]). Elevated plasma VEGF‐A was associated with incident diffusion‐weighted imaging lesions at 1‐year follow up, and lower MoCA score, in a mild ischaemic stroke cohort enriched for imaging features of SVD (*n* = 181) [89]. Higher plasma VEGF‐D was associated with increased SVD burden in a study of 64 older adults [90], and serum VEGF‐A correlated positively with cSVD score in 144 adults with mild–moderate SVD [91]. The disparity between plasma VEGF‐A in AD (lower) and SVD severity (higher) suggests that changes in CSF and blood VEGF‐A levels in AD are likely to be confounded by the presence of vascular co‐pathologies.

Plasma PlGF is emerging as a robust marker of white matter (WM) injury associated with vascular cognitive impairment. Plasma PlGF was also elevated in AD (*n* = 102) and frontotemporal lobar degeneration (FTLD) (*n* = 40) compared to non‐demented controls (*n* = 38) but was highest in subcortical ischaemic vascular dementia (SVID) (*n* = 70). Plasma PlGF correlated positively with WMH severity and inversely with cognition across the combined dementia cohort [92]. Elevated baseline PlGF correlated positively with WMH burden and inversely with WM integrity as quantified by fractional anisotropy (FA) in 272 older cognitively unimpaired adults. Baseline plasma PlGF also predicted steeper cognitive decline but was unrelated to WMH or FA trajectories [93]. In 370 over‐55‐year‐olds from the MarkVCID cohort, higher plasma PlGF was associated with higher MRI‐FW (free water), a marker of WM interstitial fluid; this association accounted for 26% of the relationship between PLGF and CDR and 73% of the relationship between PlGF and WMH [94]. In a multisite observational study, plasma PlGF was also associated with an increased odds ratio of a higher Fazekas score (OS 1.16) and clinical dementia rating (CDR) (OR 1.22) and could reliably discriminate CDR 0 from CDR 0.5 in people with Fazekas score ≥2, demonstrating the potential of plasma PlGF as a diagnostic marker for WMH [95].

PlGF levels tend to be elevated in CSF and peripheral blood in AD, particularly in patients with concomitant white matter pathology. Serum PlGF levels were elevated in AD (*n* = 109) compared to cognitively unimpaired controls (*n* = 56) and non‐AD cognitively impaired individuals (*n* = 76), and were highest in AD patients who also had WMH and cerebral microbleeds [96]. Higher plasma PlGF was associated with WMH severity, specifically in AD biomarker positive subjects from memory clinics; initial findings in a discovery cohort of 79 AD and 20 non‐AD patients were confirmed in a validation cohort of 54 AD patients [97]. PlGF levels in the CSF were closely associated with cognitive decline and CSF‐tau, particularly in PET Aβ+ve individuals with evidence of BBB leakiness, as measured in matched CSF and serum samples from cognitively unimpaired, MCI and AD participants in the ADNI (*n* = 25 per group) [98]. AD and vascular dementia share common and overlapping pathological features (reviewed [99, 100, 101]) with up to 84% of brains from elderly people having both vascular and AD pathology [102]—the presence of vascular pathology in AD is likely to contribute significantly to CSF and serum PlGF expression in AD and mixed dementia.

Limbic‐predominant age‐related TDP‐43 encephalopathy neuropathological change (LATE‐NC) may contribute to cognitive decline in ~25% of community‐dwelling cohorts [103]. LATE‐NC pathology is associated with hippocampal sclerosis (HS) and was present in ~57% of brains in AD [104]. Microvascular pathologies, including arteriolosclerosis, are highly prevalent in LATE‐NC and HS [105, 106] with over 80% of LATE‐NC brains reported to show some degree of microvascular pathology [105]. In this study, moderate–severe arteriosclerosis was observed within the basal ganglia in ~32%, in the anterior watershed region ~48%, and the posterior watershed region ~42% of cases. The extent of arteriosclerosis within the posterior watershed was predictive of LATE‐NC stage. In another large autopsy study of over ~2000 brains from the Nun Study (University of Kentucky) and National Alzheimer's Coordinating Centre, arteriolosclerosis was strongly associated with HS pathology across multiple brain regions independently of cardiovascular risk factors, including hypertension and diabetes [106]. A recent study revealed that CSF TDP‐43 level (which was inversely associated with hippocampal TDP‐43 pathology) correlated inversely with sVEGFR1 [107]. Future studies should focus on the interrelationships between VEGF‐signalling, vascular pathology, LATE‐NC, and LATE‐NC‐associated HS.

## 5 VEGF GENE EXPRESSION INTERACTS WITH APOE TO INFLUENCE COGNITIVE DECLINE

Single‐nucleotide polymorphisms (SNPs) within the VEGF‐A promoter region, particularly SNP 2578A/A and 1154A/A, are linked to AD risk. In a disease setting, SNP 2578A/A and 1154A/A are generally associated with lower serum VEGF‐A [108] (in agreement with the associations between lower serum VEGF‐A, steeper cognitive decline, and ADNC). Both SNPs are in close proximity to binding sites of transcription factors, including HIF‐1α, suggesting that these variants may influence disease risk by impairing VEGF‐A signalling in response to cerebral ischaemia. In an Italian cohort, the VEGF‐A 2578A/A variant was associated with accelerated cognitive decline and MCI‐AD conversion (particularly in *APOE* ε4 carriers) [109]. These findings were supported by a meta‐analysis by the same group [110]. However, later case–control studies [111, 112] and two subsequent meta‐analyses failed to replicate this association [113, 114] (although one reported a weak association in *APOE* ε4 non‐carriers [114]).

Genome‐wide association studies in 2012 [115] and 2016 [116] identified SNPs within the *VEGF‐A* gene that were associated with circulating VEGF‐A levels. The first study [115], in over 3500 participants from the Framingham study, identified four such SNPs (validated in two smaller independent cohorts): rs6921438, rs4416670, rs6993770, and rs10738760, which together accounted for 48% of the hereditability of serum VEGF‐A level. A later genome‐wide analysis of over 10,000 individuals from 10 cohorts identified 10 loci that accounted for 52% of circulating VEGF‐A levels. In addition to replicating some of the findings from the earlier study, this larger study also identified unique VEGF‐A related variants in gene networks related to angiogenesis and platelet activation. Epistatic interactions between a SNP within the intronic region of VEGF‐A (rs34528081), with KCBV2/VLDLR (rs2375981) and ZFPM2 (rs6993770), were associated with AD protection. These protective variants were linked to higher VEGF‐A expression.

Five other epistatic interactions were found between VEGF‐A and VEGF‐related variants identified in the previous GWAS [115, 116]. The epistatic interactions between the *VEGF‐A* gene variants were stronger in *APOE* ε4 non‐carriers. Analysis of 10 VEGF gene markers in 531 individuals from the ROSMAP study in 2020 revealed that *VEGF‐A* and *NRP1* interacted with *APOE* and that this was associated with poorer cognition [117]. *NRP1* gene expression was associated with worse cognitive outcome in *APOE* ε4 carriers but a better cognitive outcome in *APOE* ε4 non‐carriers. The findings suggest that *VEGF* gene expression influences the risk conferred by *APOE* in AD. APOE is also a ligand for the very low‐density lipoprotein receptor, which itself has also been associated with increased risk of AD.

## 6 CURRENT PERSPECTIVES AND FUTURE DIRECTION

Canonical VEGF‐A/VEGFR2 signalling is disrupted in AD. Despite elevated VEGF‐A expression, as demonstrated by biochemical and sn‐RNA analysis of human brain tissue in AD, a shift in the expression of VEGFR2 (decreased) relative to VEGFR1 (increased), sequestration of VEGF‐A by soluble VEGFR1 or Aβ, or age‐related reduction in VEGF‐A expression, is likely to impair VEGF‐A/VEGFR2‐mediated angiogenic signalling. This is associated with pathogenic angiogenesis, vascular remodelling and BBB leakiness, as shown recently in early‐stage AD [44]. VEGFR1 is induced under hypoxic and inflammatory conditions, and VEGF‐A/VEGF1 signalling may directly (via inhibition of VEGF‐A/VEGFR2 signalling) and indirectly (via activation of macrophages and microglia and the release of angiogenic mediators) promote vascular remodelling and BBB leakiness [33]. Intraperitoneal injection of VEGF‐A restored memory and reduced disease pathology, in association with the induction of angiogenesis, in APP mouse models of AD [118, 119].

Reduced VEGF‐A/VEGFR2 signalling has also been implicated in neuronal degeneration [120], probably due partly to reduced perfusion as a result of pathogenic angiogenesis and vascular remodelling, and partly due to a loss of the protective trophic effects of VEGF‐A and VEGFR2 signalling on neuronal survival, maturation, and function [121]. VEGF‐A also plays a key role in the maturation and morphology of neurons [122]. Defective VEGF‐A/VEGFR2 signalling, due to oligomeric Aβ blocking VEGFR2 receptor signalling [51], causes synaptic dysfunction, which can be rescued by overexpression of VEGF‐A in *APP/PS1* double transgenic mice [53]. VEGF‐A also interacts with Aβ and prevents fibril formation [70] and may modulate both parenchymal and vascular Aβ deposition.

**FIGURE 5:**
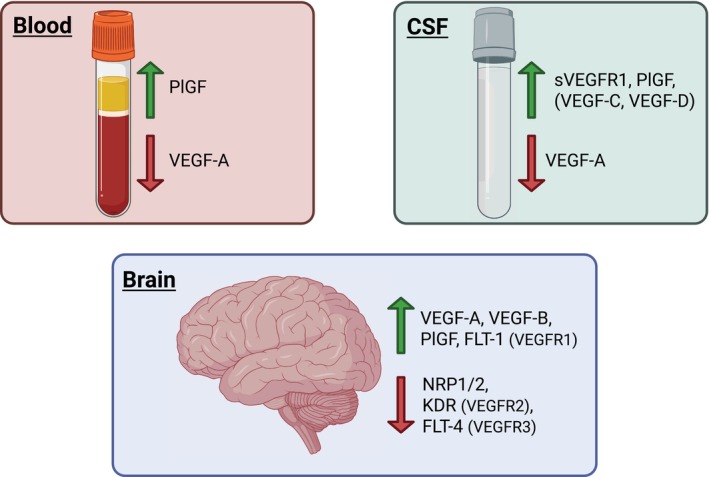
Overview of changes in VEGF signalling in Alzheimer's disease. A shift in the relative expression of KDR/VEGFR2 (reduced) and FLT‐1/VEGFR1 (increased), or sequestration of VEGF‐A by soluble VEGFR1 or Aβ, is likely to contribute to pathogenic angiogenesis and neuronal dysfunction in AD. Disruption of the VEGF‐A/VEGFR2 pathway is reflected by lower VEGF‐A in CSF and blood, and higher sVEGFR1 in CSF. VEGF‐B/FLT1 signalling genes are upregulated in microglia and associated with accelerated cognitive decline and ADNC. VEGF signalling via VEGFR2 and VEGFR3, and co‐receptors NRP1/2, are associated with better cognitive outcomes and lower ADNC, but their expression levels are reduced in AD. Red arrows = reduced level/expression of ‘protective’ VEGF pathways in AD. Green arrows = increased expression of disease‐associated pathways in AD. FLT‐1, gene for vascular endothelial growth factor receptor 1; FLT‐4, gene for vascular endothelial growth factor receptor 3; KDR, gene for vascular endothelial growth factor receptor 2; PlGF, gene for placental growth factor; VEGF‐A/B/C, genes for vascular endothelial growth factors A, B and C. Created with BioRender.com.

In summary, findings from recent multi‐omic studies, combining sn‐RNA transcriptomic and proteomic analysis in matched CSF, blood, and post‐mortem tissue across independent cohorts, and analysis of VEGF signalling proteins in large independent cohorts, have revealed that dysregulated VEGF signalling is related to cognitive decline and neuropathological change in AD. The studies reveal that VEGF signalling pathways within the central nervous system involve diverse populations of cells and extend beyond classical VEGF‐A/VEGFR2 signalling within the cerebral vasculature. Enrichment of VEGF‐B/FLT1 signalling within microglial and neuronal cells suggests an involvement of VEGF signalling in regulating microglial activation. Protective VEGF signalling pathways, including VEGF‐C signalling, and NRP1 and NRP2 expression, are reduced in AD. Lower levels of VEGF‐A in CSF and serum, and elevated VEGFR1 in CSF, are related to cognitive decline and ADNC and may be useful biomarkers to track early disease progression in AD. A summary of the major findings of recent studies is shown in Figure 5.

## 7 CONCLUSION

VEGF signalling is involved in the pathogenesis of several neurodegenerative diseases. In amyotrophic lateral sclerosis, for example, VEGF‐A levels are reduced in plasma and motor deficits are rescued by VEGF‐A administration in animal models [123]. The evidence presented in this review suggests a similar contribution of VEGF signalling in AD, with lower levels of VEGF‐A and elevated VEGFR1 in CSF and blood associated with steeper cognitive decline in Aβ‐ and tau‐positive individuals. Recent sn‐RNA studies have indicated that abnormal VEGF signalling in AD extends beyond the classical VEGF‐A/VEGFR2 pathway, involving *VEGF‐B/FLT1* signalling in microglia that induces a pro‐inflammatory response and a potential loss of protective compensatory VEGF‐C signalling involving NRP1 and NRP2, which may facilitate the clearance of Aβ. CSF and serum VEGF‐A, PlGF, and VEGFR1 have potential as biomarkers to identify and track early vascular and pathological changes in people at risk of AD. Anti‐VEGF monoclonal antibodies and pharmaceuticals that suppress endothelial VEGFR1 expression have shown promise in reducing pathology and improving cognition in rodent models of AD [47, 124]. In summary, VEGF pathways remain an intensive and active area of research in AD, underpinning the development of new diagnostic and prognostic biomarkers and potentially of novel therapeutics.

## AUTHOR CONTRIBUTIONS

Conceptualization: Cherelle E. G. Emery and J. Scott Miners. Analysis/Investigation: Cherelle E. G. Emery and J. Scott Miners. Drafting/Editing: Cherelle E. G. Emery, J. Scott Miners, and Seth Love.

## Data Availability

The data that support the findings of this study are available in Human_BBB at https://twc-stanford.shinyapps.io/human_bbb/.
